# Patient-Specific Deep Reinforcement Learning for Automatic Replanning in Head-and-Neck Cancer Proton Therapy

**Published:** 2025-08-11

**Authors:** Malvern Madondo, Yuan Shao, Yingzi Liu, Jun Zhou, Xiaofeng Yang, Zhen Tian

**Affiliations:** Department of Radiation and Cellular Oncology, University of Chicago, Chicago, IL, USA; Division of Environmental and Occupational Health Sciences, University of Illinois at Chicago, Chicago, IL, USA; Department of Radiation and Cellular Oncology, University of Chicago, Chicago, IL, USA; Department of Radiation Oncology and Winship Cancer Institute, Emory University, Atlanta, GA, USA; Department of Radiation Oncology and Winship Cancer Institute, Emory University, Atlanta, GA, USA; Department of Radiation and Cellular Oncology, University of Chicago, Chicago, IL, USA

## Abstract

Anatomical changes in head-and-neck cancer (HNC) patients during intensity-modulated proton therapy (IMPT) can shift the Bragg Peak of proton beams, risking tumor underdosing and organ-at-risk (OAR) overdosing. As a result, treatment replanning is often required to maintain clinically acceptable treatment quality. However, current manual replanning processes are often resource intensive and time consuming. In this work, we propose a patient-specific deep reinforcement learning (DRL) framework for automated IMPT replanning, with a reward-shaping mechanism based on a 150-point plan quality score designed to handle competing clinical objectives in radiotherapy planning. We formulate the planning process as a reinforcement learning (RL) problem where agents learn high-dimensional control policies to adjust plan optimization priorities to maximize plan quality. Unlike population-based approaches, our framework trains personalized agents for each patient using their planning Computed Tomography (CT) and augmented anatomies simulating anatomical changes (tumor progression and regression). This patient-specific approach leverages anatomical similarities along the treatment course, enabling effective plan adaptation. We implemented and compared two DRL algorithms, Deep Q-Network (DQN) and Proximal Policy Optimization (PPO), using dose-volume histograms (DVHs) as state representations and a 22-dimensional action space of priority adjustments. Evaluation on eight HNC patients using actual replanning CT data showed that both DRL agents improved initial plan scores from 120.78 ± 17.18 to 139.59 ± 5.50 (DQN) and 141.50 ± 4.69 (PPO), surpassing the replans manually generated by a human planner (136.32±4.79). Further comparison of dosimetric endpoints confirms these improvements translate to better tumor coverage and OAR sparing across diverse anatomical changes. This work highlights the potential of DRL in addressing the geometric and dosimetric complexities of adaptive proton therapy, offering a promising solution for efficient offline adaptation and paving the way for online adaptive proton therapy.

## 1. Introduction

Intensity-modulated proton therapy (IMPT) provides highly conformal tumor coverage while sparing surrounding organs at risk (OARs), leveraging the unique dose deposition characteristics of the proton beam’s Bragg peak (Holliday et al., 2015; McKeever et al., 2016; Moreno et al., 2019). However, the Bragg peak makes IMPT highly sensitive to interfractional anatomical changes, such as tumor progression or regression, weight loss, and edema, that alter tissue density along the beam path (Huiskes et al., 2023; Sonke et al., 2019). These anatomical changes can shift the Bragg peak, degrading tumor coverage and/or OAR sparing, and often necessitate one or multiple manual replanning sessions during the treatment course to maintain clinical quality. Addressing these anatomical changes currently requires manual intervention: a multi-step process involving patient reimaging, anatomical recontouring, and iterative, trial-and-error plan optimization. This manual adaptation workflow is notoriously labor intensive and typically takes several days, delaying optimal care and straining limited clinical resources (Kim et al., 2018; Leeman et al., 2017; Li et al., 2020).

From a machine learning (ML) perspective, automating treatment planning presents several challenges. First, the planning process involves navigating a high-dimensional optimization space with complex, non-linear relationships between planning parameters and resulting dose distributions (Burlacu et al., 2025; Wildman et al., 2024). Second, the multi-objective nature of treatment planning demands careful balancing of target coverage with the sparing of multiple OARs, each governed by its clinical constraints, thereby desiring methods that can effectively navigate trade-offs along a complex Pareto frontier (Barker Jr et al., 2004; Bobić et al., 2023; Sonke et al., 2019). Finally, the limited availability of patient data and substantial inter-patient variability in anatomy and tumor characteristics make it difficult to develop models that generalize well across large patient populations (Nikou et al., 2024; Visak et al., 2024; Volpe et al., 2021). These challenges are further amplified in head and neck cancer (HNC) treatment planning, which involves multiple treatment targets with varying prescription dose levels, and tumors often invade or abut several critical organs.

Reinforcement learning (RL) offers a compelling framework for tackling these sequential, multi-objective optimization challenges in HNC treatment planning. RL agents learn optimal strategies through interaction and reward feedback, making them particularly well-suited for navigating the complex, iterative process of balancing competing clinical objectives in treatment planning (Sutton et al., 1998). Specifically, RL holds the potential to automate the iterative decision-making process of priority adjustments in treatment planning (Eckardt et al., 2021; Ebrahimi and Lim, 2021; Moreau et al., 2021; Yang et al., 2024). Furthermore, RL’s inherent adaptability aligns with the vision of personalized online adaptive radiotherapy, where treatment plans can be dynamically adapted based on a patient’s tumor response and anatomical changes along the treatment course.

To realize this potential for automated IMPT replanning, we develop a patient-specific deep reinforcement learning (DRL) framework for the dosimetrically challenging HNC. Our approach formulates the priority tuning process during plan optimization as an RL problem, where the state is defined by dose–volume histograms (DVHs) for clinical target volumes (CTVs) and OARs, and the action space comprises 22 predefined, clinically informed priority adjustments, enabling the agent to navigate the trade-offs inherent in this multi-objective optimization problem. Plan quality is quantified via a comprehensive 150-point scoring system that combines ProKnow standardized scoring criteria (Nelms et al., 2012) with institutional planning guidelines, and the reward is defined as the change in the plan quality score. Our framework trains personalized DRL agents using each patient’s anatomy captured by the initial planning Computed Tomography (CT) images and augmented anatomies simulating anatomical variations. This patient-specific approach leverages the patient’s inherent anatomical consistency along the treatment course to optimize performance specifically for that individual rather than attempting to develop a one-size-fits-all solution.

### Generalizable Insights about Machine Learning in the Context of Healthcare

Our work offers broader insights for applying machine learning in healthcare settings characterized by high variability and limited data:

- **Patient-specific architectures merit exploration in high-variability clinical applications.** In medical settings characterized by limited data availability and substantial anatomical variability, developing ML models that generalize across large patient cohorts remains a fundamental challenge. However, despite undergoing anatomical changes, each patient typically maintains high anatomical consistency throughout the treatment course, which is a feature that has been entirely underutilized in traditional population-based ML models. By simulating plausible anatomical variations from a patient’s baseline anatomy to augment the limited patient-specific training datasets, our proposed patient-specific strategy offers a viable alternative to traditional population-based ML models and aligns closely with the goals of personalized precision medicine.
- **Clinically-informed reward design ensures alignment.** Directly incorporating established clinical planning guidelines and quantitative plan scoring metrics into the RL reward function is crucial. This ensures that learned policies optimize for objectives recognized as clinically valid and relevant to real-world clinical decision-making priorities, fostering trust and clinical utility.

## 2. Related Work

RL has increasingly shown promise for automating radiotherapy treatment planning. Early demonstrations in cervical cancer high-dose-rate brachytherapy introduced a DRL-based Virtual Treatment Planner (VTP) that emulated human planners by observing DVH inputs and adjusting parameter weights, yielding plans that outperformed both the initial and human-generated plans (Shen et al., 2019). Building on this success, the framework was extended to external beam radiotherapy for prostate cancer (Shen et al., 2020), where separate DQN subnetworks adapted parameters in a relatively simple scenario with a single target and two OARs. Subsequent efforts by the same research group focused on improving training efficiency through rule-based adjustments informed by human-planner experience (Shen et al., 2021a) and scalability through a hierarchical VTP network that decomposed the planning process into structure selection, parameter selection, and action adjustment (Shen et al., 2021b), although still limited to single-target cases with few OARs. Diverging from neural network-based approaches, Zhang et al. (2020) developed an interpretable planning bot for pancreatic stereotactic body radiation therapy using linear function approximation for Q-learning. However, these approaches were primarily designed for anatomically simpler treatment sites and might struggle with sites like HNC, characterized by complex inter-structure trade-off relationships.

HNC represents a substantially more challenging planning context due to multiple target volumes with varying prescription doses and numerous critical structures in close proximity, demanding tight dosimetric trade-offs even for experienced planners. Recent RL adaptations to HNC planning have attempted to navigate this high-dimensional optimization space with varied strategies. Gao et al. (2024) adapted the hierarchical VTP approach for HNC *photon* therapy (whereas our work focuses on *proton* therapy), employing two DQN subnetworks for parameter selection and adjustment direction determination while representing states as plan quality scores rather than DVHs. To manage the high dimensionality arising in HNC planning, they manually fixed most of the 141 potential planning parameters in their case based on dosimetrist expertise, allowing their VTP to learn adjustments for only 8 unique priority parameters. Similarly, grappling with the high-dimensional action space, Wang and Chang (2024) explored policy-gradient methods with a transformer-based PPO agent for continuous parameter adjustments. Their approach proposed dynamically reducing complexity by adjusting parameters only for the subset of structures currently exhibiting the lowest quality scores. However, this strategy may not accommodate cases where certain OARs have to be strategically compromised to maintain target coverage—a common situation in complex HNC planning scenarios.

Notably, all the aforementioned approaches rely on population-based models trained across diverse patient cohorts. While effective at capturing common anatomical patterns, such models face challenges adapting to patient-specific anatomical changes that necessitate treatment replanning during HNC’s fractionated radiotherapy (Caudell et al., 2017; Ma et al., 2022; Maniscalco et al., 2023; Visak et al., 2024). This limitation, stemming from the under-utilization of intra-patient anatomical consistency, restricts their ability to generate optimally adapted plans tailored to each patient’s unique anatomical features and evolving pattern. Motivated by these challenges, we introduce a novel patient-specific RL framework for IMPT replanning in HNC. By developing and optimizing the planning policy to each individual’s unique anatomy and dosimetric constraints from the outset, our approach inherently leverages intra-patient consistency patterns. This personalization is crucial for effectively managing the high-dimensional optimization space and is potentially better suited to addressing the adaptive replanning demands inherent in HNC treatment, overcoming key limitations of existing population-based strategies.

## 3. Methods

### 3.1. Treatment Plan Optimization

In treatment planning systems (TPS), the patient anatomy (CTVs and OARs) is discretized into voxels. The dose ${\text{d}}_{i}$ deposited in each voxel i is linearly dependent on adjustable beamlet intensities, represented by the fluence vector $\text{x}\;\in {\mathbb{R}}_{+}^{n}$. This relationship is precalculated and stored as a dose influence matrix D, where each element ${\text{D}}_{ij}$ quantifies the dose contribution from unit intensity of beamlet j to voxel i (Gorissen, 2022):

(1) $${\text{d}}_{i}\;=\;\sum_{j}D_{ij}x_{j}.$$

The goal of treatment plan optimization is to determine the optimal beamlet intensity vector x that minimizes dose deviations in CTV voxels from their prescribed dose while reducing the dose to OARs, which can be formulated as:

(2) $$\begin{array}{l} \underset{\text{x}\in {\mathbb{R}}^{n}}{\min}\;𝓛\left(\text{x}\right)\;=\;\sum_{m=1}^{M}\omega_{{\text{CTV}}_{m}}{\left\|{\text{D}}_{{\text{CTV}}_{m}}\text{x}\;-\;d_{\text{Rx,}\;{\text{CTV}}_{m}}\right\|}^{2}\;+\;\sum_{k=1}^{M}\omega_{{\text{OAR}}_{k}}{\left\|{\text{D}}_{{\text{OAR}}_{k}}\text{x}\right\|}^{2}\\ \;\;\;\;\;\;\;\text{s.t}.\;\;\;\;\;x_{j}\;\geq \;0,\;\;\;\;\;\;\text{for}\;j\;=\;\left\{1,\ldots ,n\right\}. \end{array}$$

Here, $𝓛\left(\text{x}\right)$ is the multi-objective treatment planning loss function, where $\text{x}\in {\mathbb{R}}^{n}$ is the vector of n beamlet intensities to be optimized. Each component $x_{j}$ represents the intensity or weight of the j-th beamlet. The constraint $x_{j}\;\geq \;0$ explicitly ensures that all beamlet intensities are non-negative. The formulation incorporates M CTVs, where $m\;\in \;\left\{1,\ldots ,M\right\}$ indexes each CTV. Similarly, K OARs are considered, where $k\;\in \;\left\{1,\ldots ,K\right\}$ indexes each OAR. For the patient cohort in this study, M≤3 and K≤12. ${\text{D}}_{{\text{CTV}}_{m}}$ and ${\text{D}}_{{\text{OAR}}_{k}}$ denote the dose influence matrices for the m-th CTV and k-th OAR. $d_{\text{Rx},\;{\text{CTV}}_{m}}$ is the prescribed dose vector for the m-th CTV.

The weighting parameters $\omega_{{\text{CTV}}_{m}}\;\geq \;0$ and $\omega_{{\text{OAR}}_{k}}\;\geq \;0$ serve as treatment planning priorities (TPPs) that balance competing clinical objectives: achieving prescribed dose coverage in the m-th CTV while minimizing dose exposure to the k-th OAR. While this weighted loss function is a standard proxy for the clinical objective, it does not directly optimize for the clinical constraints that human planners must satisfy (e.g., specific DVH criteria for various organs listed in Table 1). In practice, achieving perfect dose homogeneity is not feasible, and different OARs have varying dose tolerances based on their biological characteristics. These clinical realities mean that planners must manually and iteratively adjust the weighting parameters and re-solve the optimization problem until clinical requirements are met–a time-consuming and resource-intensive process.

We automate this critical step by introducing a DRL agent to perform this hyperparameter search. The agent learns to directly optimize for clinical constraint satisfaction, using a reward signal derived from the dose plan’s adherence to clinical goals. The DRL agent interacts with a plan optimization engine that serves as the core of our RL environment, minimizing the cost function (Eqn. 2) with agent-selected priority weights. This integration of dose calculation with RL-based planning optimization streamlines a major bottleneck in the radiotherapy workflow.

### 3.2. Reinforcement Learning for Automatic Replanning

Converting the aforementioned priority tuning during inverse plan optimization into an RL problem offers a solution to the time-consuming, labor-intensive manual adjustment process. We model the priority tuning as a finite Markov Decision Process defined by the tuple (𝓢, 𝓐, 𝓟, 𝓡, γ), where:

- **State Space** $\left(𝓢\right)$: Each state $s_{t}\;\in \;𝓢$ represents the DVHs of the 3 CTVs and 12 typical OARs (Table 1), represented as a normalized M×N matrix where M corresponds to anatomical structures and N to discretized dose-volume bins.
- **Action Space** $\left(𝓐\right)$: A discrete set of 22 clinically constrained actions representing priority adjustments for different planning objectives, designed based on clinical experience. Adjustment details are provided in Appendix C.
- **Transition Dynamics** $\left(𝓟\right)$: The transition model $𝓟\;:\;𝓢\;\times \;𝓐\;\times \;𝓢\;\to \;\left[0,\;1\right]$ is governed by a treatment optimization engine, with $𝓟\left(s_{t+1}|s_{t},\;a_{t}\right)$ capturing how priority adjustment actions affect the resulting dose distribution.
- **Reward Function** $\left(𝓡\right)$: The reward function 𝓡:𝓢×𝓐→ℝ defines the immediate reward obtained after taking action $a_{t}$ in state $s_{t}$. It is defined as $r\left(s_{t},\;a_{t}\right)\;=\;\psi \left(s_{t}\;+\;1\right)-\;\psi \left(s_{t}\right)$, where $\psi \left(\cdot \right)$ is the plan quality score of the intermediate plan dose, generated by taking action $a_{t}$. We designed a 150-point scoring system based on the standardized ProKnow scoring criteria (Nelms et al., 2012) and institution-specific planning guidelines. It includes a set of dosimetric metrics for the structures of interest. The final score was calculated as the sum of the scores across all these metrics, with higher scores indicating better plan quality. The plot of the scoring function for each dosimetric metric is presented in Appendix D.
- **Discount Factor** $\left(\gamma \right)$: γ∈[0,1) balances immediate dosimetric improvements with long-term overall plan quality.

The goal is to learn an optimal policy $\pi^{*}$ that dictates the sequence of priority adjustments leading to the highest possible cumulative reward, thereby yielding high-quality treatment plans:

$$\pi^{*}\;=\;\arg\underset{\pi }{\max}E\left[\sum_{t=0}^{T}\gamma^{t}r\left(s_{t},\;a_{t}\right)\;|\;s_{0},\;\pi \right]$$

where π:𝓢→𝓐 represents a policy that maps DVH states to priority adjustment actions, and T is the planning horizon. The expectation E is taken over the stochasticity in state transitions under policy π starting from initial state $s_{0}$.

The optimal policy formulation provides a theoretical objective, but solving for $\pi^{*}$ directly is challenging in high-dimensional state spaces like those encountered in IMPT replanning. To address this challenge, we implemented two state-of-the-art DRL algorithms that have demonstrated success in complex sequential decision-making tasks.

#### Deep Q-Network (DQN):

In Q-learning, the objective is to learn the optimal action-value function $Q^{*}\left(s,a\right)$, which represents the maximum expected return achievable by taking action a in state s and following the optimal policy thereafter (Sutton et al., 1998; Watkins and Dayan, 1992). For proton therapy planning, this corresponds to predicting which priority adjustment $\left(a\right)$ will yield the greatest long-term improvement in plan quality. The optimal Q-function satisfies the Bellman equation:

$$Q^{*}\left(s,a\right)\;=\;E_{s^{\prime }∼P\left(\cdot |s.a\right)}\left[r\left(s,\;a\right)\;+\;\gamma \;\max a^{\prime }Q^{*}\left(s^{\prime },\;a^{\prime }\right)\right]$$

DQN approximates this optimal Q-function using a deep neural network $Q\left(s,a;\;\text{θ}\right)$ with weights θ. The network is trained by minimizing a loss function that measures the temporal difference (TD) error between the predicted Q-value and the target Q-value (Mnih et al., 2015). The loss function at iteration i is given by:

(3) $${𝓛}_{\text{DQN,}i}\left({\text{θ}}_{i}\right)\;=\;E_{\left(s,a,r,s^{\prime }\right)∼𝓑}\left[{\left(y_{i}\;-\;Q\left(s,a;\;{\text{θ}}_{\text{i}}\right)\right)}^{2}\right]$$

where 𝓑 is a replay buffer storing past experiences $\left(s,a,r,s^{\prime }\right)$ (often with prioritized sampling based on TD error), and the target $y_{i}$ is calculated using a separate target network $Q\left(s^{\prime },a^{\prime };\;{\text{θ}}_{i}^{-}\right)$ with delayed weights ${\text{θ}}_{i}^{-}$, typically updated periodically:

$$y_{i}\;=\;r\;+\;\gamma \max\;a^{\prime }Q\left(s^{\prime },a^{\prime };\;{\text{θ}}_{i}^{-}\right)$$

The DQN agent learns a policy by selecting actions with the highest Q-value for a given state (e.g., using an ϵ-greedy strategy to balance exploration and exploitation).

#### Proximal Policy Optimization (PPO):

While DQN learns Q-values of state-action pairs and derives policies through action selection mechanisms, PPO takes a more direct approach. PPO is an actor-critic policy gradient algorithm that explicitly learns a policy $\pi ((a|s;\;{\text{θ}}_{\text{p}})$ parameterized by weights ${\text{θ}}_{\text{p}}$ and a value function $V(s;{\text{θ}}_{\text{v}})$ parameterized by weights ${\text{θ}}_{\text{v}}$. The policy (actor) determines actions based on the current state, while the value function (critic) approximates the value of the current state, providing a baseline for evaluating the actor’s performance. Policy gradient methods update the actor’s parameters by following the gradient of an objective function that aims to maximize the expected return (Schulman et al., 2017). To ensure stable training, PPO employs a clipped surrogate objective function that prevents excessively large policy updates by maintaining the new policy within a trust region of the old policy. The objective function for the actor is defined as:

$$L_{\text{actor}}\;\text{=}\;E_{(s,a)∼\pi_{{\text{θ}}_{{\text{p}}_{\text{old}}}}}\left[\min\left(\frac{\pi_{{\text{θ}}_{\text{p}}}(a|s)}{\pi_{{\text{θ}}_{{\text{p}}_{\text{old}}}}(a|s)}{\hat{A}}_{t}(s,a),\text{clip}\left(\frac{\pi_{{\text{θ}}_{\text{p}}}(a|s)}{\pi_{{\text{θ}}_{{\text{p}}_{\text{old}}}}(a|s)},\;1\;-\;\epsilon ,\;1\;\text{+}\;\epsilon \right){\hat{A}}_{t}(s,a)\right)\right]$$

where $\pi_{{\text{θ}}_{{\text{p}}_{\text{old}}}}$ is the policy from the previous iteration, $\pi_{{\text{θ}}_{\text{p}}}$ is the current policy, ϵ is a hyperparameter controlling the clipping range, and ${\hat{A}}_{t}(s,a)$ is the generalized advantage estimate (GAE) (Schulman et al., 2016):

(4) $${\hat{A}}_{t}(s_{t},a_{t})\;\text{=}\;\sum_{l\text{=}0}^{T-t-1}{\left(\gamma \lambda \right)}^{l}(r_{t+l}\;\text{+}\;\gamma V_{{\text{θ}}_{\text{v}}}(s_{t+l\text{+}1})\;-\;V_{{\text{θ}}_{\text{v}}}(s_{t+l}))$$

where γ∈[0,1) is the discount factor, λ∈[0,1] controls the bias-variance tradeoff, and $V_{{\text{θ}}_{\text{v}}}$ represents the value function approximation. GAE estimates how much better an action is compared to the average action in a given state.

Simultaneously, the critic is trained to accurately estimate the state value by minimizing the mean squared error between its predictions and the actual discounted return:

$$L_{\text{critic}}\;=\;E_{\left(s_{t},R_{t}\right)∼𝓑}[{\left(V_{{\text{θ}}_{\text{v}}}\left(s_{t}\right)-R_{t}\right)}^{2}]$$

where $R_{t}\;=\;\sum_{k=0}^{T-t}\gamma^{k}r_{t+k}$ is the discounted return and 𝓑 is the experience replay of the current policy.

In essence, PPO aims to find optimal policy and value function parameters by iteratively minimizing a combined objective function that includes both the actor loss and the critic loss, thereby approximately solving the minimization problem given by:

(5) $$L_{\text{PPO}}\;=\;\underset{{\text{θ}}_{\text{p}},{\text{θ}}_{\text{v}}}{\min}E_{\left(s,a\right)∼𝓑}\left[-L_{\text{actor}}\left({\text{θ}}_{\text{p}}\right)\;+\;c_{1}L_{\text{critic}}\left({\text{θ}}_{\text{v}}\right)-\;c_{2}E_{s}\left[H\left(\pi_{{\text{θ}}_{\text{p}}}\left(.|s\right)\right)\right]\right]$$

where $c_{1}$ and $c_{2}$ are coefficients controlling the relative importance of the value function loss and the entropy bonus $H\left(\cdot \right)$, respectively. The entropy bonus encourages exploration.

## 4. Patient Cohort

### 4.1. Cohort Selection

We validated our approach using a retrospective cohort of 18 HNC patients treated with IMPT at the Emory Proton Therapy Center. Eight patients requiring replanning due to substantial anatomical changes were used for patient-specific RL training, while the remaining 10 patients served as training data for population-based RL baselines. For each patient, an initial planning CT (pCT) was acquired 1 − 2 weeks before the treatment course, and a replanning CT (rpCT) was obtained when significant anatomical changes were observed and warranted replanning. Performance evaluation used all eight rpCT cases for both RL approaches. These cases were selected for their varied tumor characteristics and spatial relationships to surrounding OARs, all involving three CTVs representing challenging planning scenarios. While the primary CTV (CTV1) for all cases was prescribed 70 GyRBE over 35 fractions, the prescription doses for the secondary (CTV2) and tertiary (CTV3) targets varied per case (Table 2). This prescription heterogeneity, combined with substantial inter-patient anatomical variation, poses a major challenge for automated treatment planning in HNC, particularly for population-based models struggling to generalize across heterogeneous clinical objectives. Patient demographics and staging are detailed in Table 6 (Appendix B).

### 4.2. Data Extraction

Relevant patient data, including the pCT and rpCT, and their corresponding CTV and OAR contours, were retrieved from an internal database in DICOM format. All the contours were manually delineated by attending physicians for treatment planning or replanning of the actual treatments. These DICOM files of CT images and contours were imported into the open-source treatment planning toolkit matRad (Wieser et al., 2017) to precalculate dose influence matrices (D) required for plan optimization, using the same beam arrangements (e.g., isocenter location, number of beams, and beam angles) as those used in the actual treatments. The dose matrix calculated from the pCT and its associated contours was used for training the DRL agents, while the matrix calculated from the rpCT and corresponding contours was used for testing.

To train a patient-specific agent capable of adapting treatment plans to anatomical changes, we generated augmented anatomies by simulating tumor variations. In this proof-of-concept study, we simulated only two variation scenarios, i.e., tumor progression and regression, by expanding the original CTV contours on the pCT by 2 mm or shrinking them by 3 mm, respectively. These variations not only changed the CTV volumes but also altered their spatial relationships with surrounding OARs, increasing the diversity of the patient-specific training dataset in terms of anatomy and planning complexity. The pCT images, along with each set of modified contours, were also imported into matRad to calculate the corresponding dose influence matrices for the simulated anatomy variations.

## 5. Experiments & Results

In this section, we present the results of our experiments and evaluate the effectiveness of RL-based planners for automated IMPT replanning in HNC treatment.

### 5.1. Experimental Setup

For each patient, both DQN and PPO agents were trained using the patient’s original anatomy from the pCT and two augmented anatomical variations. The state representation included the normalized DVH curves for all structures $\text{(dim}\;\text{=}\;\left(\text{15,100}\right)\text{)}$, while the discrete action space consisted of 22 priority adjustments for CTVs and OARs. Each agent was trained for 100 episodes, with each episode consisting of up to 15 priority adjustment steps or terminating early if the maximum plan quality score of 150 was achieved. Algorithm 1 in Appendix A provides a detailed overview of this DRL-based replanning process, illustrating the integration of priority tuning and plan optimization within our experimental framework. Details of network specifications and hyperparameters are provided in Appendix E.

The trained agents were then tested on the patient’s new anatomy captured in the rpCT. Specifically, starting from an initial default priority set ${\text{ω}}_{0}$, the agents performed priority tuning following the same episodic framework described above, where each step involved selecting a tuning action based on the intermediate plan’s quality, followed by inverse plan optimization. The number of tuning attempts (horizon length) in this process can be extended as needed, bounded only by computational cost. As a comparative baseline, a human planner also generated a new manual treatment plan for each case, employing manual priority tuning. The performance of the agents was assessed by comparing the plan quality scores and dosimetric metrics of the agent-generated plans with those of the manually created plans.

To facilitate the interaction between the RL agents and the treatment planning process, we developed a custom OpenAI Gymnasium-compatible environment that simulates the treatment planning workflow. At each time step, the environment receives the agent-selected action at $a_{t}$ and applies it to modify the current priority weights ω. Using these updated priorities along with the dose influence matrices ${\text{D}}_{\text{CTV}m}$ and ${\text{D}}_{{\text{OAR}}_{k}}$ for each CTVm and OARk, the environment solves the optimization problem (Eqn. 2) via projected gradient descent (Fu et al., 2023; Ghobadi et al., 2012; Nocedal and Wright, 1999) to update the beamlet weights x and generate a new dose distribution d (Eqn. 1). The resulting dose distribution is evaluated against clinical constraints (Table 1) to compute the plan score and reward (Appendix D). Each action $a\;\in \;\left\{0,\;1,\;\ldots ,\;21\right\}$ updates priority weights ω according to

(6) $$\begin{array}{cc} \omega_{\left(t+1\right)}\;=\;{\left[\omega_{\left(t\right)}\;+\;\Delta_{a}\right]}_{0}^{1}, & t\;\in \;\left[0,\;14\right], \end{array}$$

where ${\left[x\right]}_{0}^{1}\;≜\;\min\left(\max\left(x,\;0\right),\;1\right)$ ensures weights remain within valid bounds. The details of the priority adjustment $\Delta_{a}$ are included in the Appendix C. All experiments were conducted on a workstation equipped with an NVIDIA RTX 5000 Ada Generation GPU with 32 GB of memory.

### 5.2. Plan Quality Comparisons

The same 150-point scoring system used to calculate rewards during DRL training was employed to quantify the plan quality of treatment plans generated for each patient’s new anatomy captured in the rpCT. To provide comprehensive benchmarking, we evaluated our patient-specific approach against both manual planning and population-based RL baselines. The population-based agents were trained on the initial planning CTs of the remaining 10 patients in our cohort using identical architecture, hyperparameters, and training setup, consistent with conventional clinical practice where models are trained on population data.

The resulting plan quality scores on the 1^st^ replanning CT are presented in Table 3, with detailed dosimetric metrics summarized in Appendix F. All RL approaches effectively adjusted planning objective priorities, significantly improving plan quality compared to initial plans generated using default priority sets. Crucially, patient-specific PPO achieved the highest mean quality score (141.50 ± 4.69), outperforming manual plans (136.32 ± 4.79, *p* < 0.01 via paired t-test), population-based PPO (139.17 ± 4.83), patient-specific DQN(139.59 ± 5.50), and population-based DQN (134.52 ± 8.64). Furthermore, the patient-specific PPO-generated plans exhibited lower score variability (σ=4.69), indicating enhanced consistency and reliability in adapting to anatomical changes compared to all other methods.

Overall, patient-specific agents matched or exceeded manual plans in all cases, with PPO achieving the highest score in 7 out of 8 patients. Beyond manual benchmarks, patient-specific agents significantly outperformed population-based agents in 14 of the 16 comparisons (across all patients and algorithms), underscoring the clear advantages of personalized adaptation over generalized models, especially when handling unique anatomical changes.

### 5.3. Dosimetric Analysis

**Figure 1:**
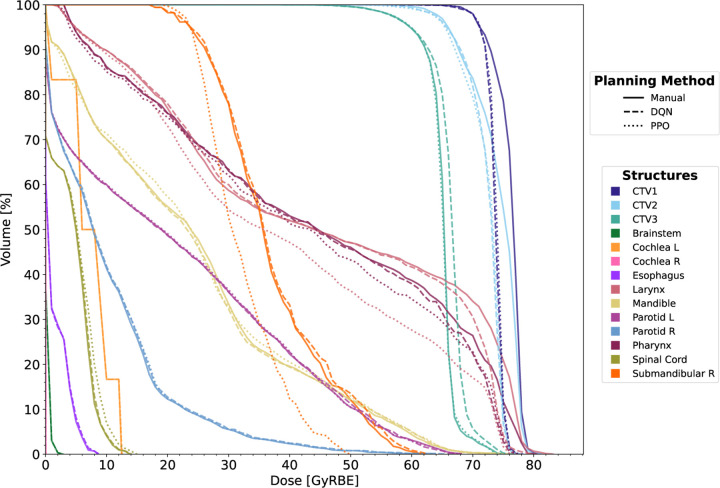
DVH comparison for patient P4’s 1^st^ replanning CT: Manual replans (solid lines), patient-specific DQN (dashed lines), and patient-specific PPO (dotted lines). Complete DVH curves and dosimetric analysis for all patients’ 1^st^ rpCTs are provided in Appendix F.

Detailed dosimetric metrics for each structure on the 1^st^ replanning CT, comparing manually generated plans with those from patient-specific DQN and PPO agents, are presented in Table 4 (P1-P5) and Table 18 (P6-P8, Appendix F). Clinically acceptable target dose coverage was achieved in all the three plans for every case, with the coverage of primary $\text{CTV}\;\left(V_{d_{Rx,{\text{CTV}}_{1}}}\right)$ no less than 97.97%. In terms of dose homogeneity inside the primary $\text{CTV}\left(\text{CTV}1\right)$, both RL agents reduced hot spots in three patients while maintaining excellent coverage. Most notably, in patient P4, DQN and PPO reduced the maximum dose within CTV1 by 3.41 GyRBE and 2.65 GyRBE, respectively, compared to the manual plan. PPO demonstrated superior OAR sparing in most patients, reducing mean dose to the larynx (LAR) 36.15 ± 3.38 GyRBE (PP O) vs. 38.48 ± 5.76 GyRBE (Manual) and to the bilateral parotids (PARL and PARR) 19.81 ± 5.86 GyRBE (PPO) vs. 22.00 ± 8.66 GyRBE (Manual) , compared to manual replanning. The effectiveness of PPO was particularly evident in case P5, where it slightly compromised the dose coverage of the tertiary CTV (CTV3, 97.53%) to substantially reduce the dose to the right submandibular gland (SMGR), bringing its mean dose closer 37.60 GyRBE to the dose tolerance (35 GyRBE) and resulting in a significantly higher plan score. In contrast, the manual and DQN-generated plans for this case substantially exceeded the dose tolerance of the SMGR, with mean doses of 67.28 GyRBE and 70.89 GyRBE, respectively. Figure 1 compares the DVH curves for patient P4, illustrating results from manually generated plans and those produced by patient-specific RL agents (DQN and PPO).

### 5.4. Adaptation to Inter-fractional Changes

To further assess the robustness of our approaches in handling rapidly evolving anatomies, we extended our comparison to include the second replanning CT for cases in our evaluation cohort that underwent multiple replanning sessions during the treatment course (P1, P4, P6, P7, P8). As shown in Table 5, patient-specific RL demonstrated superior adaptation capabilities, achieving better performance than population-based baselines in 9 out of 10 metric comparisons (DQN: 4/5 cases; PPO: 5/5 cases). Patient-specific DQN, in particular, achieved a higher mean score of 138.43±3.91 compared to 127.78±12.14 for the population-based DQN baseline, while showing markedly lower variability. Although population-based approaches showed efficacy in handling certain cases with common anatomical patterns (e.g., P4 with DQN), the patient-specific approach consistently demonstrated better adaptation to the unique inter-fractional changes of individual patients.

## 6. Discussion

Our study demonstrates that patient-specific RL-based automated IMPT replanning, particularly using PPO, consistently generates treatment plans of higher quality for HNC patients experiencing anatomical changes during the treatment course, compared to both manually generated plans and population-based RL approaches. These improvements have several important implications for adaptive proton therapy workflows.

### 6.1. Clinical Significance

The marked improvement in plan quality scores across all patients indicates that patient-specific RL-based automated replanning can standardize high-quality treatment planning tailored to individual anatomical variations. This personalized approach may lead to reduced treatment-related toxicities while maintaining effective tumor control. For example, the reduction in parotid gland dose has been correlated with decreased risk of xerostomia, with studies suggesting that each 1 GyRBE reduction in mean dose translates to approximately 4% reduction in xerostomia risk (Castelli et al., 2015; Chao et al., 2001). As shown in Table 4, patients P1 and P2 benefited from substantial parotid sparing (reductions of 10.7 GyRBE and 3.3 GyRBE, respectively), while patients P1 and P4 experienced notable laryngeal sparing (reductions of 3.6 GyRBE and 6.0 GyRBE).

Importantly, these dosimetric improvements were achieved without compromising target coverage, underscoring the ability of patient-specific RL methods to address the geometric and dosimetric complexities unique to each patient’s HNC anatomy and balance complex trade-offs to achieve clinically acceptable treatment plans. The superior performance of patient-specific agents over population-based approaches (14/16 comparisons in Table 3) demonstrates that individualized adaptation is crucial for handling the substantial inter-patient anatomical variability characteristic of HNC. The proposed patient-specific RL-based automated replanning approach offers a promising solution for efficient offline adaptation and paves the way for personalized online adaptive proton therapy.

### 6.2. RL-based Planning and Patient-specific Benefits

Patient-specific RL algorithms outperformed both manual replanning and population-based counterparts, with PPO consistently delivering superior plans compared to DQN. This finding aligns with recent literature suggesting policy-based methods like PPO effectively navigate the complex reward landscapes common in radiotherapy planning (Wang and Chang, 2024). PPO’s strategy of performing small, incremental parameter updates helps prevent detrimental large policy shifts, a feature particularly well-suited to patient-specific radiotherapy optimization where individual anatomical nuances require careful navigation.

The advantage of patient-specific adaptation becomes evident when analyzing replanning outcomes relative to individual anatomical features (Table 2). On the 1^st^ replanning CT (Table 3), patient-specific PPO achieved the highest plan scores in 7/8 cases, outperforming manual replans across diverse target volumes (rpCT CTV1 range: 69.63–297.74 cc) and volumetric changes between pCT and rpCT (e.g., P2 CTV3: +15.0% expansion). This superiority is highlighted by its achievement of the highest absolute score (148.77) for the patient with the smallest CTV1/CTV2 (P4) and clinically meaningful gains for large-volume cases over manual plans (e.g., patient P3: +7.08; P5: +2.83). Furthermore, the lower score variability of patient-specific approaches (σ=4.69 for PPO) suggests more consistent adaptation to each patient’s unique anatomical constraints.

This performance gap widens on the 2^nd^ replanning CT (Table 5), where the benefits of patient-specific DRL become even more pronounced. Both patient-specific DQN and PPO agents consistently and significantly outperformed their population-based counterparts. Patient-specific PPO, in particular, achieved the highest overall mean score (PPO: 139.22 ± 4.65 vs. PPO-popn: 134.50 ± 6.91). In contrast, the high score variability observed in population-based approaches (σ=12.14 for DQN-popn) demonstrates that these models struggle to generalize and consistently adapt to the diverse and dynamic anatomical changes. This confirms that the benefits of patient-specific training persist and amplify as anatomical changes accumulate throughout the treatment course.

These findings collectively demonstrate that patient-specific DRL delivers significant dosimetric advantages over population-averaged approaches by learning and adapting to individual patient characteristics. By consistently delivering high-quality plans tailored to each patient’s evolving anatomy, this framework holds considerable promise for improving adaptive radiotherapy outcomes in HNC treatment.

### 6.3. Limitations and Future Directions

While our findings demonstrate the feasibility of deep reinforcement learning (DRL) for adaptive IMPT replanning, important limitations should be acknowledged alongside opportunities for future work. First, the retrospective evaluation was conducted on a limited cohort of eight patients. However, this cohort is representative of challenging HNC cases with three CTVs of different prescription levels and common anatomical change patterns. Future work should apply our patient-specific approach to a larger, more diverse cohort of patients with varying CTV configurations, rigorously assessing the framework’s adaptability across different anatomical presentations. The demonstrated superiority of our patient-specific DRL agents over manual plans further motivates comprehensive evaluation in prospective studies.

Second, the current framework’s reliance on simulated anatomical changes, while methodologically necessary for controlled RL development, necessitates validation with real longitudinal anatomical changes observed during actual treatment courses. Simulated target volume expansions and contractions may not fully capture the complex, heterogeneous tissue deformations, weight loss patterns, tumor shrinkage dynamics, and normal tissue responses that occur during radiotherapy. Future work should incorporate actual or additional anatomical change scenarios for training to enhance the performance of the DRL agents further.

Finally, methodological advancements to the RL agent itself could yield further dosimetric improvements. While our current approach focuses on clinically interpretable discrete priority adjustments, exploring continuous action spaces could potentially allow for finer control over planning parameters and enable more nuanced treatment planning. Exploring more sophisticated state representations may further reduce computational demands and enhance plan quality. To address the computational overhead associated with patient-specific training, transfer learning techniques might be needed. Such techniques could facilitate the development of readily deployable models, requiring only minimal fine-tuning for new patients.

## 7. Conclusion

Reinforcement learning, particularly PPO-based approaches, offers a compelling approach to automated replanning in HNC IMPT. The patient-specific nature of our RL framework enables tailored optimization strategies that adapt to the unique anatomical and dosimetric challenges of each patient. Our findings demonstrate a consistent generation of superior treatment plans compared to manual planning, potentially reducing planning time and improving plan quality. These results suggest that RL-based solutions can significantly enhance IMPT workflows, ultimately benefiting cancer patients through reduced toxicities and effective tumor control.

## Figures and Tables

**Figure 2:**
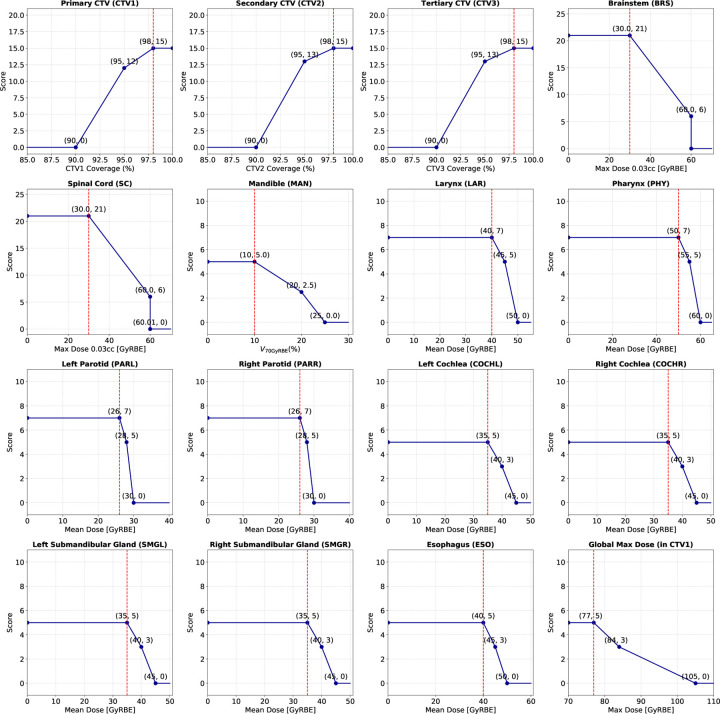
Scoring functions for different dosimetric parameters. ProKnow-based scoring functions for quantifying the quality of treatment plans. The vertical red-dashed lines denote clinical planning goals. Plan quality is the sum of all components (maxscore=150.0).

**Table 1:** **Planning objectives of IMPT inverse plan optimization for HNC**, including dose-volume constraints for CTVs and OARs. Here, $V_{d}$ is the volume receiving at least dose d, and $D_{v}$ is the minimum dose received by the hottest v volume of a structure.

Structure	Planning Objective
CTV1 (primary CTV)	$V_{d_{Rx,{\text{CTV}}_{\text{1}}}}\;\geq \;98\%$ of CTV1 volume $D_{0\%}\;\leq \;110\%$ of $d_{Rx,{\text{CTV}}_{\text{1}}}$
CTV2 (secondary CTV)	$V_{d_{Rx,{\text{CTV}}_{2}}}\;\geq \;98\%$ of CTV2 volume
CTV3 (tertiary CTV)	$V_{d_{Rx,{\text{CTV}}_{3}}}\;\geq \;98\%$ of CTV3 volume
Brainstem (BRS)	$D_{0.03\text{cc}}\;\leq \;30$ GyRBE
Spinal Cord (SC)	$D_{0.03\text{cc}}\;\leq \;30$ GyRBE
Mandible (MAN)	$V_{70\text{GyRBE}}\;\leq \;10\%$ of MAN volume
Larynx (LAR)	$D_{\text{mean}}\;\leq \;45$ GyRBE
Pharynx (PHY)	$D_{\text{mean}}\;\leq \;50$ GyRBE
Left Parotid (PARL)	$D_{\text{mean}}\;\leq \;26$ GyRBE
Right Parotid (PARR)	$D_{\text{mean}}\;\leq \;26$ GyRBE
Left Cochlea (COCHL)	$D_{\text{mean}}\;\leq \;35$ GyRBE
Right Cochlea (COCHR)	$D_{\text{mean}}\;\leq \;35$ GyRBE
Left Submandibular Gland (SMGL)	$D_{\text{mean}}\;\leq \;35$ GyRBE
Right Submandibular Gland (SMGR)	$D_{\text{mean}}\;\leq \;35$ GyRBE
Esophagus (ESO)	$D_{\text{mean}}\;\leq \;40$ GyRBE

*Note*: GyRBE = dose (Gy) × relative biological effectiveness (RBE, typically 1.1 for protons).

**Table 2:** Treatment Parameters and CTV Volumetric Changes. Prescription doses, replanning frequency, and CTV volumes (cc) on planning CT (pCT) and replan CTs (rpCT) for primary CTV (CTV1), secondary CTV (CTV2), and tertiary CTV (CTV3). All patients received 35 fractions. NA indicates no second replan was performed.

Case ID	Prescription (GyRBE)			No. of Replans	Volume on pCT (cc)			Volume on 1^st^ rpCT (cc)			Volume on 2^nd^ rpCT (cc)		
	CTV1	CTV2	CTV3		CTV1	CTV2	CTV3	CTV1	CTV2	CTV3	CTV1	CTV2	CTV3
P1	70.00	59.85	53.90	2	182.58	441.98	199.84	150.30	386.54	196.90	133.45	362.06	182.75
P2	70.00	63.00	56.00	1	182.65	419.51	55.78	172.37	403.60	64.11	NA	NA	NA
P3	70.00	59.85	53.90	1	206.38	522.20	40.53	184.04	453.84	35.69	NA	NA	NA
P4	70.00	63.00	56.00	2	84.40	122.69	219.46	69.63	106.29	217.84	65.78	102.78	195.47
P5	70.00	63.00	56.00	1	211.17	362.35	485.46	194.52	346.14	476.32	NA	NA	NA
P6	70.00	60.20	53.90	2	242.93	246.37	50.93	297.74	253.30	52.11	180.45	232.65	55.72
P7	70.00	63.00	56.00	2	122.19	263.02	440.24	132.33	267.06	442.82	120.17	255.05	434.99
P8	70.00	59.85	53.90	2	89.09	229.30	185.22	83.10	213.64	171.83	77.36	213.06	174.36

**Table 3:** Plan quality scores on 1^st^ replanning CT (0–150 scale; higher preferred). Comparison of treatment plans generated using the initial default priority set (referred to as *initial*), manually created plans (*manual*), and plans automatically generated by both population-based(*-popn*) and patient-specific DQN and PPO agents. Bold values denote the highest score for each patient.

Case ID	Initial	Manual	DQN*-popn*	DQN	PPO*-popn*	PPO
P1	122.56	130.85	130.40	131.05	137.01	**137.88**
P2	83.14	132.09	135.33	136.20	135.64	**138.05**
P3	132.35	140.44	144.17	147.25	147.31	**147.52**
P4	132.14	143.94	148.38	146.10	146.38	**148.77**
P5	132.96	138.67	138.41	138.32	138.30	**141.50**
P6	125.65	133.03	128.87	**135.24**	135.18	135.18
P7	108.67	132.53	126.27	141.03	136.51	**141.35**
P8	128.75	139.03	124.31	141.53	137.06	**141.78**
**mean** ± **std**	120.78 ± 17.18	136.32 ± 4.79	134.52 ± 8.64	139.59 ± 5.50	139.17 ± 4.83	**141**.**50** ± **4**.**69**

**Table 4:** Summary of dosimetric performance across patients P1-P5 on 1^st^ rpCT: Manual (M), patient-specific DQN (Q), and patient-specific PPO (P). All dose metrics $\left(D_{\text{0\%/0.03cc/mean}}\right)$ in GyRBE. Bold values indicate superior dosimetry outcomes. Results for P6-P8 in Table 18 (Appendix F).

Structure	Metric	P1			P2			P3			P4			P5		
		M	Q	P	M	Q	P	M	Q	P	M	Q	P	M	Q	P
CTV1	$V_{d_{Rx,{\text{CTV}}_{\text{1}}}}\;\geq \;98\%$	97.99	**98.01**	97.99	97.99	**98.01**	97.99	97.99	**98.00**	97.99	**98.01**	97.97	**98.01**	97.99	97.99	**98.00**
	$D_{0\%}\;\leq \;77$	81.86	**81.02**	82.24	**82.30**	83.03	83.61	81.52	81.00	**79.68**	81.23	**77.82**	78.58	**77.26**	80.82	77.82
CTV2	$V_{d_{Rx,{\text{CTV}}_{2}}}\;\geq \;98\%$	97.97	97.99	**98.56**	97.94	**98.27**	98.04	**98.39**	**98.39**	98.29	**98.55**	98.14	98.19	**99.51**	98.50	99.19
CTV3	$V_{d_{Rx,{\text{CTV}}_{3}}}\;\geq \;98\%$	97.84	98.03	**99.00**	97.76	**98.27**	98.18	98.18	**98.33**	98.02	98.02	97.94	**98.03**	**99.12**	97.98	97.53
BRS	$D_{0.03\text{cc}}\;\leq \;30$	**23.29**	24.01	24.17	19.59	**19.58**	20.04	10.69	**10.55**	10.77	2.87	**2.82**	2.85	13.69	**12.48**	14.07
SC	$D_{0.03\text{cc}}\;\leq \;30$	**37.63**	37.67	40.60	**22.63**	22.70	22.85	**31.81**	32.35	32.17	**13.85**	14.04	14.98	30.58	**30.23**	32.06
MAN	$V_{70\text{GyRBE}}\;\leq \;10\%$	1.73	**0.58**	2.18	1.29	**1.07**	1.51	0.13	**0.00**	1.28	0.22	**0.09**	**0.09**	**0.08**	0.67	0.20
LAR	$D_{\text{mean}}\;\leq \;40$	43.76	43.84	**40.15**	36.51	**36.48**	36.76	**34.76**	34.84	34.91	45.29	44.51	**39.29**	32.10	32.74	**30.86**
PHY	$D_{\text{mean}}\;\leq \;50$	**0.00**	**0.00**	**0.00**	49.13	**48.95**	49.41	**0.00**	**0.00**	**0.00**	44.00	43.17	**42.13**	37.73	38.98	**35.12**
PARL	$D_{\text{mean}}\;\leq \;26$	**14.43**	14.57	15.56	26.00	26.00	**22.74**	20.39	**20.19**	20.52	**21.76**	21.79	21.86	10.04	**9.63**	10.12
PARR	$D_{\text{mean}}\;\leq \;26$	34.60	34.50	**23.86**	28.41	**25.15**	25.81	32.68	25.97	**25.71**	**10.08**	10.28	10.19	**21.56**	21.81	21.69
COCHL	$D_{\text{mean}}\;\leq \;35$	4.03	4.15	**3.96**	39.34	39.82	**33.55**	**0.02**	**0.02**	**0.02**	**6.92**	6.93	6.97	**0.00**	**0.00**	**0.00**
COCHR	$D_{\text{mean}}\;\leq \;35$	2.09	**2.07**	2.42	**8.44**	8.47	8.56	**0.84**	0.88	**0.84**	**0.02**	**0.02**	**0.02**	**0.00**	**0.00**	**0.00**
SMGL	$D_{\text{mean}}\;\leq \;35$	**59.73**	60.59	60.07	72.57	**72.11**	72.68	**0.00**	**0.00**	**0.00**	**0.00**	**0.00**	**0.00**	**68.14**	71.57	68.15
SMGR	$D_{\text{mean}}\;\leq \;35$	**0.00**	**0.00**	**0.00**	72.83	**72.51**	73.06	**0.00**	**0.00**	**0.00**	36.87	37.30	**31.98**	67.28	70.89	**37.60**
ESO	$D_{\text{mean}}\;\leq \;40$	**11.09**	**11.09**	11.57	**6.23**	6.26	6.24	**13.26**	13.35	13.31	**1.36**	1.38	1.38	6.41	**5.85**	6.69
**Plan Score**	**(max=150):**	130.85	131.05	**137.88**	132.09	136.20	**138.05**	140.44	147.25	**147.52**	143.94	146.10	**148.77**	138.67	138.32	**141.50**

*Note*: OAR abbreviations - BRS: Brainstem, SC: Spinal Cord, MAN: Mandible, LAR: Larynx, PHY: Pharynx, PARL/PARR: Left/Right Parotid, COCHL/COCHR: Left/Right Cochlea, SMLG/SMGR: Left/Right Submandibular Gland, ESO: Esophagus.

**Table 5:** Population-based(*-popn*) vs. patient-specific DRL performance on 2^nd^ replanning CT: Comparison of DQN and PPO approaches for patients requiring multiple replanning sessions. Plan quality scores (0–150 scale; higher preferred).

Case ID	DQN*-popn*	DQN	PPO*-popn*	PPO
P1	124.95	133.33	126.78	**133.64**
P4	**146.23**	143.13	145.11	145.88
P6	132.55	135.69	135.16	**136.54**
P7	115.27	140.57	135.21	**141.11**
P8	119.92	**139.42**	130.26	138.92
**mean** ± **std**	127.78 ± 12.14	138.43 ± 3.91	134.50 ± 6.91	**139**.**22** ± **4**.**65**

**Figure 3:**
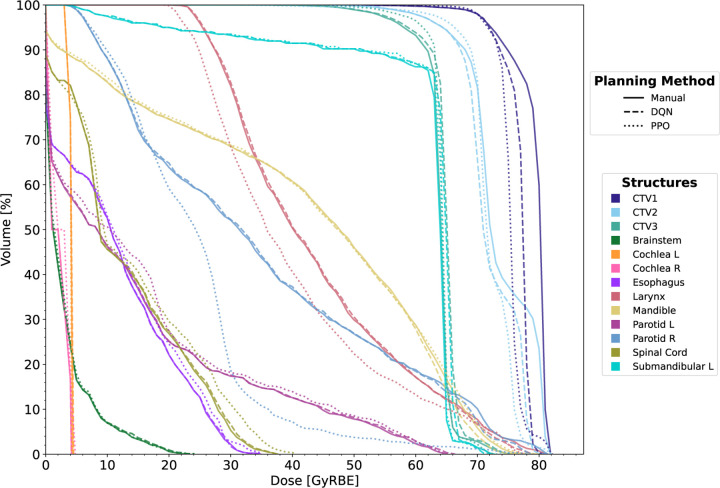
DVH comparison for patient P1’s 1^st^ rpCT: Manual replans (solid lines), patient-specific DQN (dashed lines), and patient-specific PPO (dotted lines).

**Figure 4:**
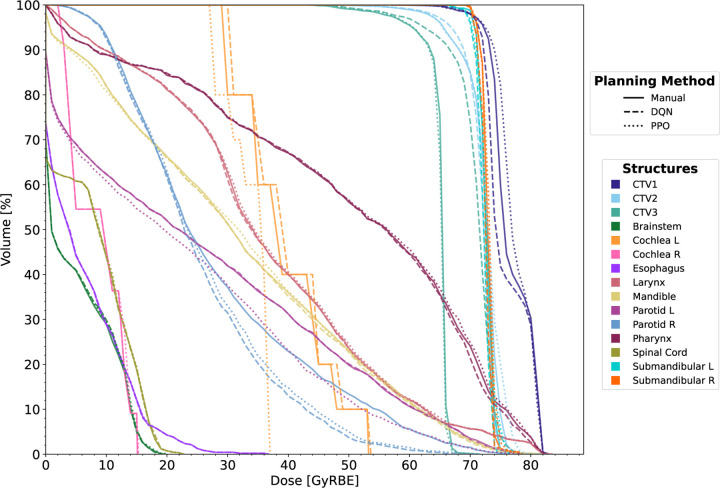
DVH comparison for patient P2’s 1^st^ rpCT: Manual replans (solid lines), patient-specific DQN (dashed lines), and patient-specific PPO (dotted lines).

**Figure 5:**
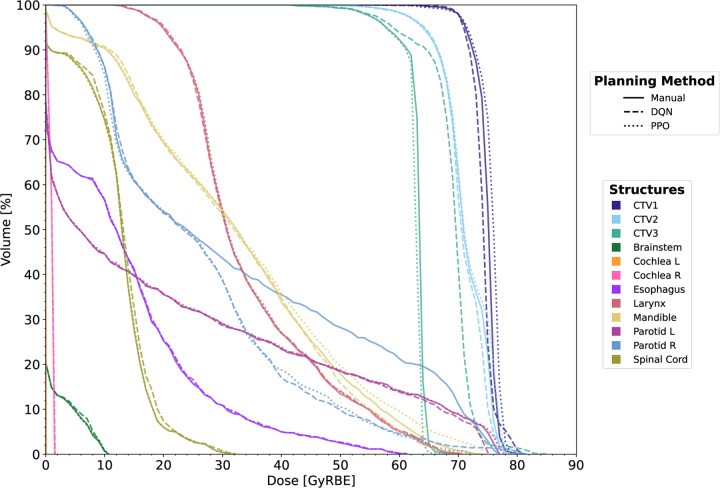
DVH comparison for patient P3’s 1^st^ rpCT: Manual replans (solid lines), patient-specific DQN (dashed lines), and patient-specific PPO (dotted lines).

**Figure 6:**
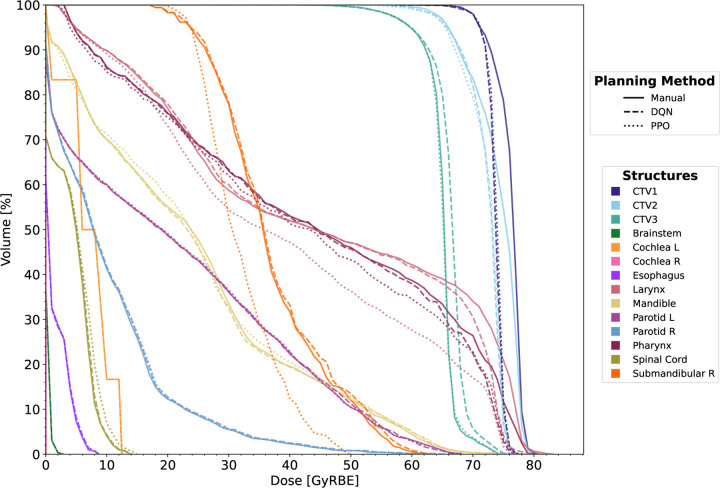
DVH comparison for patient P4’s 1^st^ rpCT: Manual replans (solid lines), patient-specific DQN (dashed lines), and patient-specific PPO (dotted lines).

**Figure 7:**
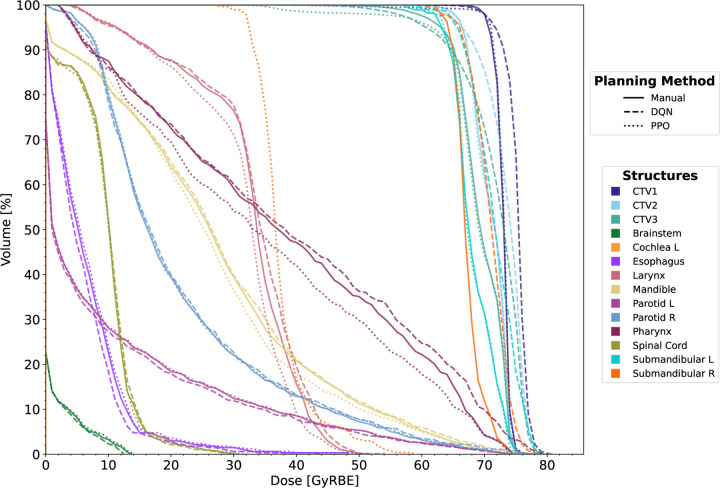
DVH comparison for patient P5’s 1^st^ rpCT: Manual replans (solid lines), patient-specific DQN (dashed lines), and patient-specific PPO (dotted lines).

**Figure 8:**
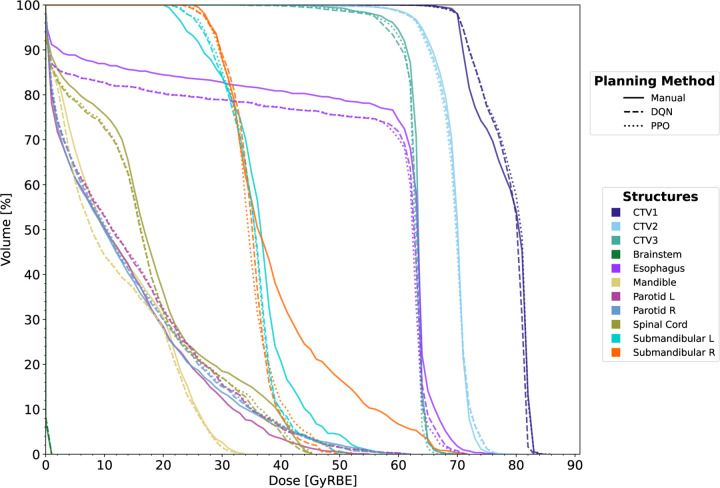
DVH comparison for patient P6’s 1^st^ rpCT: Manual replans (solid lines), patient-specific DQN (dashed lines), and patient-specific PPO (dotted lines).

**Figure 9:**
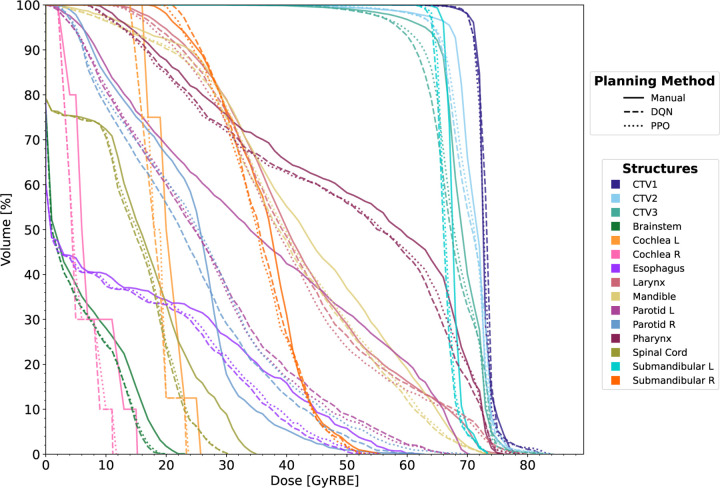
DVH comparison for patient P7’s 1^st^ rpCT: Manual replans (solid lines), patient-specific DQN (dashed lines), and patient-specific PPO (dotted lines).

**Figure 10:**
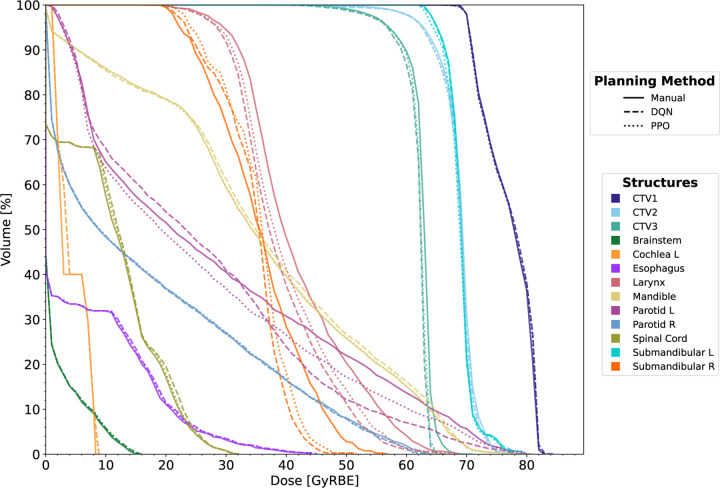
DVH comparison for patient P8’s 1^st^ rpCT: Manual replans (solid lines), patient-specific DQN (dashed lines), and patient-specific PPO (dotted lines).

## Appendix

### Appendix B. Cohort Demographics and Staging

Table 6 summarizes the demographic and clinical staging data for the eight patients included in this study. Patient ages range from 48 to 73 years. The cohort encompasses a range of disease stages, including stage II (n=2), stage III (n=3), stage IVa (n=1), stage IVb (n=1), and one unassigned case. Detailed TNM classifications are also reported, reflecting the heterogeneity of tumor and nodal involvement across the sample.

**Table 6:** Patient Demographics and Staging Information.

Case ID	Age	Overall Stage	TNM Stage
P1	69	IVb	T0 N3 M0
P2	73	Unassigned	T4 & T2 N2 M0
P3	60	II	T3 N2 M0
P4	55	III	T4 N0 M0
P5	48	II	T2 N2 M0
P6	72	IVa	T4 N0 M0
P7	71	III	T4 N0 M0
P8	59	III	T4 N1 M0

### Appendix C. Priority Adjustments: Structure-Specific Modifications

All replanning agents modify the treatment plan optimization priorities through 22 discrete adjustments to weight parameters. The complete mapping is detailed in Table 7, organized by structure type and adjustment magnitude. This discrete action space enables clinically meaningful trade-off adjustments while maintaining valid priority ranges. For critical OARs (brainstem, spinal cord), larger adjustments (+0.3) are available to prioritize their protection, while other structures have more moderate adjustments. Target volumes include both positive and negative adjustments to allow for both improved coverage and compromises when necessary for OAR sparing.

### Appendix D. Plan Quality Metrics

Plan quality was quantified using a comprehensive 150-point scoring system derived from standardized ProKnow criteria (Nelms et al., 2012) and institutional planning guidelines. This total score is the sum of individual component scores (detailed in Figure 2), which directly relate to the planning objectives outlined in Table 1. For reinforcement learning, the reward signal was defined as the change in this cumulative plan quality score between tuning steps.

### Appendix E. Network Architectures & Training Parameters

Both DRL agents process the DVHs using Convolutional Neural Networks (CNNs). This architectural choice is motivated by the ability of CNNs to learn meaningful spatial features and correlations from the structured DVH input, capturing complex dose-volume relationships across different anatomical structures. Common elements in both architectures include ReLU activation functions, batch normalization layers following convolutions, max-pooling for dimensionality reduction, and dropout layers for regularization. The DQN agent employs two convolutional layers followed by three fully connected layers to estimate Q-values for 22 discrete actions. The DQN implementation features experience replay with prioritized sampling based on temporal difference errors, exponential epsilon-greedy decay, and a delayed target network updated every 10 steps to stabilize learning. The PPO agent adopts a shared actor-critic architecture with a three-layer CNN backbone. The actor head outputs 22 discrete priority adjustments via a categorical distribution, while the critic estimates state values. Training incorporates clipped policy updates (ϵ=0.2), generalized advantage estimation (GAE, λ=0.95), value loss coefficient $\left(c_{1}\;=\;1.00\right)$, and entropy coefficient $\left(c_{2}\;=\;0.01\right)$ to balance exploration and exploitation. Orthogonal initialization was applied to weights in the PPO network, with constant initialization for biases. Both algorithms use the Adam optimizer with learning rate 1e−5, with PPO additionally employing gradient clipping (maxnorm=0.5) to ensure training stability. Table 8 details the architectural specifications for both models. Table 9 highlights the key hyperparameters and optimization settings used in training.

**Table 7:** Priority Adjustment Action Space. Each action modifies the weight of a specific structure by a predefined increment.

Action Index	Structure	Parameter	Adjustment $\left(\Delta_{a}\right)$
*Target Volume Adjustments*			
0, 1	CTV1	$\omega_{\text{CTV1}}$	+0.2, −0.1
2, 3	CTV2	$\omega_{\text{CTV2}}$	+0.2, −0.1
4, 5	CTV3	$\omega_{\text{CTV3}}$	+0.2, −0.1
18, 19	CTV2	$\omega_{a}$	+0.1, −0.1
20, 21	CTV3	$\omega_{b}$	+0.1, −0.1
*Organ-at-Risk Adjustments*			
6	Mandible (MAN)	$\omega_{\text{MAN}}$	+0.1
7	Brainstem (BRS)	$\omega_{\text{BRS}}$	+0.3
8	Spinal Cord (SC)	$\omega_{\text{SC}}$	+0.3
9, 10	Parotids (PARR, PARL)	$\omega_{\text{PAR(L/R)}}$	+0.1
11	Larynx (LAR)	$\omega_{\text{LAR}}$	+0.1
12	Pharynx (PHY)	$\omega_{\text{PHY}}$	+0.1
13, 14	Cochleas (COCHL, COCHR)	$\omega_{\text{COCH(L/R)}}$	+0.1
15, 16	Submand. Glands (SMGL, SMGR)	$\omega_{\text{SMG(L/R)}}$	+0.1
17	Esophagus (ESO)	$\omega_{\text{ESO}}$	+0.1

### Appendix F. Complete Dosimetric Results

This section contains the complete patient-specific results on the 1^st^ replanning CT, including DVH comparisons and detailed dosimetric metrics for the manual and DRL-based plans.

**Table 8:** Network architectures for DQN and PPO agents.

Component	DQN	PPO
Input Dimension	15 × 100 (Structures × Dose Bins)	
Input Channels	1	1
Convolutional Layers	2	3
Kernel Sizes	(3,5)	(3,5)
Padding	(1,2)	(1,2)
Pooling	2×2 MaxPool	2×2 MaxPool
BatchNorm	Yes	Yes
Dropout Rate	0.2	0.2
Hidden Dim (CNN)	32 → 64	32 → 64 → 64
Fully Connected	256 → 128 → 22	512 → 22 (Actor)
Output Activation	Linear	Softmax (Actor)

**Table 9:** Training Parameters for DQN and PPO Agents.

Parameter	DQN	PPO
Optimizer	Adam	Adam
Learning Rate	1e-5	1e-5
Minibatch Size	16	16
Discount Factor $\left(\gamma \right)$	0.99	0.99
Loss Function	MSE	Clipped Surrogate + Value MSE + Entropy Bonus
	(Eqn. 3)	(Eqn. 5)
Experience Buffer	Prioritized Replay	On-policy Rollouts
Buffer Retention	Long-term	Episodic
Epsilon-Greedy Exploration	Yes (exponential decay)	No
Advantage Estimation	–	GAE (λ=0.95)
Clipped Policy Update	–	0.2
Value Clip	–	0.2
Value Loss Coefficient $\left(c_{1}\right)$	–	1.0
Entropy Coefficient $\left(c_{2}\right)$	–	0.01
Gradient Clipping	*Optional*	0.5 (max norm)

**Table 10:** Comparison of dosimetric endpoints across planning strategies for patient P1 on 1^st^ rpCT. All dose values $\left(D_{\text{0\%/0.03cc/mean}}\right)$ are in GyRBE.

Delineated	Dosimetric Endpoint		Manual		DQN		PPO	
Structure	Metric	Score	Value	Score	Value	Score	Value	Score
CTV1	$V_{d_{Rx,{\text{CTV}}_{\text{1}}}}\;\geq \;98\%$	15	97.99	14.99	98.01	15.00	97.99	14.99
	$D_{max}\;\leq \;77$	5	81.86	3.31	81.02	3.43	82.24	3.25
CTV2	$V_{d_{Rx,{\text{CTV}}_{2}}}\;\geq \;98\%$	15	97.97	14.98	97.99	14.99	98.56	15.00
CTV3	$V_{d_{Rx,{\text{CTV}}_{3}}}\;\geq \;98\%$	15	97.84	14.89	98.03	15.00	99.00	15.00
BRS	$D_{0.03\text{cc}}\;\leq \;30$	21	23.29	21.00	24.01	21.00	24.17	21.00
SC	$D_{0.03\text{cc}}\;\leq \;30$	21	37.63	17.18	37.67	17.17	40.60	15.70
MAN	$V_{70\text{GyRBE}}\;\leq \;10\%$	5	1.73	5.00	0.58	5.00	2.18	5.00
LAR	$D_{\text{mean}}\;\leq \;40$	7	43.76	5.50	43.84	5.46	40.15	6.94
PHY	$D_{\text{mean}}\;\leq \;50$	7	0.00	7.00	0.00	7.00	0.00	7.00
PARL	$D_{\text{mean}}\;\leq \;26$	7	14.43	7.00	14.57	7.00	15.56	7.00
PARR	$D_{\text{mean}}\;\leq \;26$	7	34.60	0.00	34.50	0.00	23.86	7.00
COCHL	$D_{\text{mean}}\;\leq \;35$	5	4.03	5.00	4.15	5.00	3.96	5.00
COCHR	$D_{\text{mean}}\;\leq \;35$	5	2.09	5.00	2.07	5.00	2.42	5.00
SMGL	$D_{\text{mean}}\;\leq \;35$	5	59.73	0	60.59	0	60.07	0
SMGR	$D_{\text{mean}}\;\leq \;35$	5	0.00	5.00	0.00	5.00	0.00	5.00
ESO	$D_{\text{mean}}\;\leq \;40$	5	11.09	5.00	11.09	5.00	11.57	5.00
**Cumulative**	**Total**	**150**	**–**	130.85	**–**	131.05	**–**	**137.88**

**Table 11:** Comparison of dosimetric endpoints across planning strategies for patient P2 on 1^st^ rpCT. All dose values $\left(D_{\text{0\%/0.03cc/mean}}\right)$ are in GyRBE.

Delineated	Dosimetric Endpoint		Manual		DQN		PPO	
Structure	Metric	Score	Value	Score	Value	Score	Value	Score
CTV1	$V_{d_{Rx,{\text{CTV}}_{\text{1}}}}\;\geq \;98\%$	15	97.99	14.99	98.01	15.00	97.99	14.99
	$D_{max}\;\leq \;77$	5	82.30	3.24	83.03	3.14	83.61	3.06
CTV2	$V_{d_{Rx,{\text{CTV}}_{2}}}\;\geq \;98\%$	15	97.94	14.96	98.27	15.00	98.04	15.00
CTV3	$V_{d_{Rx,{\text{CTV}}_{3}}}\;\geq \;98\%$	15	97.76	14.84	98.27	15.00	98.18	15.00
BRS	$D_{0.03\text{cc}}\;\leq \;30$	21	19.59	21.00	19.58	21.00	20.04	21.00
SC	$D_{0.03\text{cc}}\;\leq \;30$	21	22.63	21.00	22.70	21.00	22.85	21.00
MAN	$V_{70\text{GyRBE}}\;\leq \;10\%$	5	1.29	5.00	1.07	5.00	1.51	5.00
LAR	$D_{\text{mean}}\;\leq \;40$	7	36.51	7.00	36.48	7.00	36.76	7.00
PHY	$D_{\text{mean}}\;\leq \;50$	7	49.13	7.00	48.95	7.00	49.41	7.00
PARL	$D_{\text{mean}}\;\leq \;26$	7	26.00	7.00	26.00	7.00	22.74	7.00
PARR	$D_{\text{mean}}\;\leq \;26$	7	28.41	2.78	25.15	7.00	25.81	7.00
COCHL	$D_{\text{mean}}\;\leq \;35$	5	39.34	3.27	39.82	3.07	33.55	5.00
COCHR	$D_{\text{mean}}\;\leq \;35$	5	8.44	5.00	8.47	5.00	8.56	5.00
SMGL	$D_{\text{mean}}\;\leq \;35$	5	72.57	0	72.11	0	72.68	0
SMGR	$D_{\text{mean}}\;\leq \;35$	5	72.83	0	72.51	0	73.06	0
ESO	$D_{\text{mean}}\;\leq \;40$	5	6.23	5.00	6.26	5.00	6.24	5.00
**Cumulative**	**Total**	**150**	**–**	132.09	**–**	136.20	**–**	**138.05**

**Table 12:** Comparison of dosimetric endpoints across planning strategies for patient P3 on 1^st^ rpCT. All dose values $\left(D_{\text{0\%/0.03cc/mean}}\right)$ are in GyRBE.

Delineated	Dosimetric Endpoint		Manual		DQN		PPO	
Structure	Metric	Score	Value	Score	Value	Score	Value	Score
CTV1	$V_{d_{Rx,{\text{CTV}}_{\text{1}}}}\;\geq \;98\%$	15	97.99	14.99	98.00	15.00	97.99	14.99
	$D_{max}\;\leq \;77$	5	81.52	3.35	81.00	3.43	79.68	3.62
CTV2	$V_{d_{Rx,{\text{CTV}}_{2}}}\;\geq \;98\%$	15	98.39	15.00	98.39	15.00	98.29	15.00
CTV3	$V_{d_{Rx,{\text{CTV}}_{3}}}\;\geq \;98\%$	15	98.18	15.00	98.33	15.00	98.02	15.00
BRS	$D_{0.03\text{cc}}\;\leq \;30$	21	10.69	21.00	10.55	21.00	10.77	21.00
SC	$D_{0.03\text{cc}}\;\leq \;30$	21	31.81	20.09	32.35	19.82	32.17	19.91
MAN	$V_{70\text{GyRBE}}\;\leq \;10\%$	5	0.13	5.00	0.00	5.00	1.28	5.00
LAR	$D_{\text{mean}}\;\leq \;40$	7	34.76	7.00	34.84	7.00	34.91	7.00
PHY	$D_{\text{mean}}\;\leq \;50$	7	0.00	7.00	0.00	7.00	0.00	7.00
PARL	$D_{\text{mean}}\;\leq \;26$	7	20.39	7.00	20.19	7.00	20.52	7.00
PARR	$D_{\text{mean}}\;\leq \;26$	7	32.68	0.00	25.97	7.00	25.71	7.00
COCHL	$D_{\text{mean}}\;\leq \;35$	5	0.02	5.00	0.02	5.00	0.02	5.00
COCHR	$D_{\text{mean}}\;\leq \;35$	5	0.84	5.00	0.88	5.00	0.84	5.00
SMGL	$D_{\text{mean}}\;\leq \;35$	5	0.00	5.00	0.00	5.00	0.00	5.00
SMGR	$D_{\text{mean}}\;\leq \;35$	5	0.00	5.00	0.00	5.00	0.00	5.00
ESO	$D_{\text{mean}}\;\leq \;40$	5	13.26	5.00	13.35	5.00	13.31	5.00
**Cumulative**	**Total**	**150**	**–**	140.44	**–**	147.25	**–**	**147.52**

**Table 13:** Comparison of dosimetric endpoints across planning strategies for patient P4 on 1^st^ rpCT. All dose values $\left(D_{\text{0\%/0.03cc/mean}}\right)$ are in GyRBE.

Delineated	Dosimetric Endpoint		Manual		DQN		PPO	
Structure	Metric	Score	Value	Score	Value	Score	Value	Score
CTV1	$V_{d_{Rx,{\text{CTV}}_{\text{1}}}}\;\geq \;98\%$	15	98.01	15.00	97.97	14.98	98.01	15.00
	$D_{max}\;\leq \;77$	5	81.23	3.40	77.82	3.88	78.58	3.77
CTV2	$V_{d_{Rx,{\text{CTV}}_{2}}}\;\geq \;98\%$	15	98.55	15.00	98.14	15.00	98.19	15.00
CTV3	$V_{d_{Rx,{\text{CTV}}_{3}}}\;\geq \;98\%$	15	98.02	15.00	97.94	14.96	98.03	15.00
BRS	$D_{0.03\text{cc}}\;\leq \;30$	21	2.87	21.00	2.82	21.00	2.85	21.00
SC	$D_{0.03\text{cc}}\;\leq \;30$	21	13.85	21.00	14.04	21.00	14.98	21.00
MAN	$V_{70\text{GyRBE}}\;\leq \;10\%$	5	0.22	5.00	0.09	5.00	0.09	5.00
LAR	$D_{\text{mean}}\;\leq \;40$	7	45.29	3.30	44.51	5.20	39.29	7.00
PHY	$D_{\text{mean}}\;\leq \;50$	7	44.00	7.00	43.17	7.00	42.13	7.00
PARL	$D_{\text{mean}}\;\leq \;26$	7	21.76	7.00	21.79	7.00	21.86	7.00
PARR	$D_{\text{mean}}\;\leq \;26$	7	10.08	7.00	10.28	7.00	10.19	7.00
COCHL	$D_{\text{mean}}\;\leq \;35$	5	6.92	5.00	6.93	5.00	6.97	5.00
COCHR	$D_{\text{mean}}\;\leq \;35$	5	0.02	5.00	0.02	5.00	0.02	5.00
SMGL	$D_{\text{mean}}\;\leq \;35$	5	0.00	5.00	0.00	5.00	0.00	5.00
SMGR	$D_{\text{mean}}\;\leq \;35$	5	36.87	4.25	37.30	4.08	31.98	5.00
ESO	$D_{\text{mean}}\;\leq \;40$	5	1.36	5.00	1.38	5.00	1.38	5.00
**Cumulative**	**Total**	**150**	**–**	143.94	**–**	146.10	**–**	**148.77**

**Table 14:** Comparison of dosimetric endpoints across planning strategies for patient P5 on 1^st^ rpCT. All dose values $\left(D_{\text{0\%/0.03cc/mean}}\right)$ are in GyRBE.

Delineated	Dosimetric Endpoint		Manual		DQN		PPO	
Structure	Metric	Score	Value	Score	Value	Score	Value	Score
CTV1	$V_{d_{Rx,{\text{CTV}}_{\text{1}}}}\;\geq \;98\%$	15	97.99	14.99	97.99	14.99	98.00	15.00
	$D_{max}\;\leq \;77$	5	77.26	3.96	80.82	3.45	77.82	3.88
CTV2	$V_{d_{Rx,{\text{CTV}}_{2}}}\;\geq \;98\%$	15	99.51	15.00	98.50	15.00	99.19	15.00
CTV3	$V_{d_{Rx,{\text{CTV}}_{3}}}\;\geq \;98\%$	15	99.12	15.00	97.98	14.99	97.53	14.69
BRS	$D_{0.03\text{cc}}\;\leq \;30$	21	13.69	21.00	12.48	21.00	14.07	21.00
SC	$D_{0.03\text{cc}}\;\leq \;30$	21	30.58	20.71	30.23	20.88	32.06	19.97
MAN	$V_{70\text{GyRBE}}\;\leq \;10\%$	5	0.08	5.00	0.67	5.00	0.20	5.00
LAR	$D_{\text{mean}}\;\leq \;40$	7	32.10	7.00	32.74	7.00	30.86	7.00
PHY	$D_{\text{mean}}\;\leq \;50$	7	37.73	7.00	38.98	7.00	35.12	7.00
PARL	$D_{\text{mean}}\;\leq \;26$	7	10.04	7.00	9.63	7.00	10.12	7.00
PARR	$D_{\text{mean}}\;\leq \;26$	7	21.56	7.00	21.81	7.00	21.69	7.00
COCHL	$D_{\text{mean}}\;\leq \;35$	5	0.00	5.00	0.00	5.00	0.00	5.00
COCHR	$D_{\text{mean}}\;\leq \;35$	5	0.00	5.00	0.00	5.00	0.00	5.00
SMGL	$D_{\text{mean}}\;\leq \;35$	5	68.14	0	71.57	0	68.15	0
SMGR	$D_{\text{mean}}\;\leq \;35$	5	67.28	0	70.89	0	37.60	0
ESO	$D_{\text{mean}}\;\leq \;40$	5	6.41	5.00	5.85	5.00	6.69	5.00
**Cumulative**	**Total**	**150**	**–**	138.67	**–**	138.32	**–**	**141.50**

**Table 15:** Comparison of dosimetric endpoints across planning strategies for patient P6 on 1^st^ rpCT. All dose values $\left(D_{\text{0\%/0.03cc/mean}}\right)$ are in GyRBE.

Delineated	Dosimetric Endpoint		Manual		DQN		PPO	
Structure	Metric	Score	Value	Score	Value	Score	Value	Score
CTV1	$V_{d_{Rx,{\text{CTV}}_{\text{1}}}}\;\geq \;98\%$	15	98.00	15.00	98.01	15.00	98.00	15.00
	$D_{max}\;\leq \;77$	5	84.54	2.92	84.74	2.89	85.85	2.74
CTV2	$V_{d_{Rx,{\text{CTV}}_{2}}}\;\geq \;98\%$	15	98.69	15.00	98.41	15.00	98.51	15.00
CTV3	$V_{d_{Rx,{\text{CTV}}_{3}}}\;\geq \;98\%$	15	98.46	15.00	98.15	15.00	98.39	15.00
BRS	$D_{0.03\text{cc}}\;\leq \;30$	21	1.11	21.00	1.03	21.00	1.23	21.00
SC	$D_{0.03\text{cc}}\;\leq \;30$	21	45.13	13.44	45.38	13.31	45.43	13.29
MAN	$V_{70\text{GyRBE}}\;\leq \;10\%$	5	0.00	5.00	0.00	5.00	0.00	5.00
LAR	$D_{\text{mean}}\;\leq \;40$	7	0.00	7.00	0.00	7.00	0.00	7.00
PHY	$D_{\text{mean}}\;\leq \;50$	7	0.00	7.00	0.00	7.00	0.00	7.00
PARL	$D_{\text{mean}}\;\leq \;26$	7	13.20	7.00	14.66	7.00	14.80	7.00
PARR	$D_{\text{mean}}\;\leq \;26$	7	13.67	7.00	14.13	7.00	14.31	7.00
COCHL	$D_{\text{mean}}\;\leq \;35$	5	0.00	5.00	0.00	5.00	0.00	5.00
COCHR	$D_{\text{mean}}\;\leq \;35$	5	0.00	5.00	0.00	5.00	0.00	5.00
SMGL	$D_{\text{mean}}\;\leq \;35$	5	36.19	4.53	34.94	5.00	34.97	5.00
SMGR	$D_{\text{mean}}\;\leq \;35$	5	39.59	3.16	35.01	5.00	34.96	5.00
ESO	$D_{\text{mean}}\;\leq \;40$	5	52.75	0.00	49.92	0.04	49.69	0.15
**Cumulative**	**Total**	**150**	**–**	133.03	**–**	**135.24**	**–**	135.18

**Table 16:** Comparison of dosimetric endpoints across planning strategies for patient P7 on 1^st^ rpCT. All dose values $\left(D_{\text{0\%/0.03cc/mean}}\right)$ are in GyRBE.

Delineated	Dosimetric Endpoint		Manual		DQN		PPO	
Structure	Metric	Score	Value	Score	Value	Score	Value	Score
CTV1	$V_{d_{Rx,{\text{CTV}}_{\text{1}}}}\;\geq \;98\%$	15	98.01	15.00	97.99	14.99	97.99	14.99
	$D_{max}\;\leq \;77$	5	78.64	3.77	82.44	3.22	84.30	2.96
CTV2	$V_{d_{Rx,{\text{CTV}}_{2}}}\;\geq \;98\%$	15	97.63	14.76	96.98	14.32	96.86	14.24
CTV3	$V_{d_{Rx,{\text{CTV}}_{3}}}\;\geq \;98\%$	15	98.08	15.00	97.16	14.44	97.30	14.53
BRS	$D_{0.03\text{cc}}\;\leq \;30$	21	23.07	21.00	19.85	21.00	20.23	21.00
SC	$D_{0.03\text{cc}}\;\leq \;30$	21	34.99	18.50	30.29	20.85	30.27	20.87
MAN	$V_{70\text{GyRBE}}\;\leq \;10\%$	5	0.03	5.00	0.07	5.00	0.21	5.00
LAR	$D_{\text{mean}}\;\leq \;40$	7	42.36	6.05	41.64	6.34	40.61	6.76
PHY	$D_{\text{mean}}\;\leq \;50$	7	50.47	6.81	48.31	7.00	48.50	7.00
PARL	$D_{\text{mean}}\;\leq \;26$	7	35.68	0.00	25.86	7.00	25.61	7.00
PARR	$D_{\text{mean}}\;\leq \;26$	7	23.26	7.00	23.10	7.00	25.32	7.00
COCHL	$D_{\text{mean}}\;\leq \;35$	5	20.38	5.00	18.11	5.00	18.43	5.00
COCHR	$D_{\text{mean}}\;\leq \;35$	5	7.54	5.00	5.31	5.00	5.60	5.00
SMGL	$D_{\text{mean}}\;\leq \;35$	5	67.71	5.00	66.30	0.00	66.60	0.00
SMGR	$D_{\text{mean}}\;\leq \;35$	5	35.91	4.63	35.35	4.86	34.84	5.00
ESO	$D_{\text{mean}}\;\leq \;40$	5	14.48	5.00	12.24	5.00	12.65	5.00
**Cumulative**	**Total**	**150**	**–**	132.53	**–**	141.03	**–**	**141.35**

**Table 17:** Comparison of dosimetric endpoints across planning strategies for patient P8 on 1^st^ rpCT. All dose values $\left(D_{\text{0\%/0.03cc/mean}}\right)$ are in GyRBE.

Delineated	Dosimetric Endpoint		Manual		DQN		PPO	
Structure	Metric	Score	Value	Score	Value	Score	Value	Score
CTV1	$V_{d_{Rx,{\text{CTV}}_{\text{1}}}}\;\geq \;98\%$	15	97.99	14.99	97.99	14.99	98.02	15.00
	$D_{max}\;\leq \;77$	5	82.89	3.16	84.47	2.93	83.44	3.08
CTV2	$V_{d_{Rx,{\text{CTV}}_{2}}}\;\geq \;98\%$	15	97.86	14.91	98.06	15.00	97.99	14.99
CTV3	$V_{d_{Rx,{\text{CTV}}_{3}}}\;\geq \;98\%$	15	97.77	14.85	97.51	14.68	97.51	14.68
BRS	$D_{0.03\text{cc}}\;\leq \;30$	21	15.29	21.00	15.94	21.00	15.74	21.00
SC	$D_{0.03\text{cc}}\;\leq \;30$	21	31.87	20.06	32.14	19.93	31.85	20.07
MAN	$V_{70\text{GyRBE}}\;\leq \;10\%$	5	0.46	5.00	0.52	5.00	0.46	5.00
LAR	$D_{\text{mean}}\;\leq \;40$	7	41.84	6.26	38.99	7.00	39.90	7.00
PHY	$D_{\text{mean}}\;\leq \;50$	7	0.00	7.00	0.00	7.00	0.00	7.00
PARL	$D_{\text{mean}}\;\leq \;26$	7	28.00	5.00	25.98	7.00	25.86	7.00
PARR	$D_{\text{mean}}\;\leq \;26$	7	17.32	7.00	17.20	7.00	17.31	7.00
COCHL	$D_{\text{mean}}\;\leq \;35$	5	4.15	5.00	4.40	5.00	4.37	5.00
COCHR	$D_{\text{mean}}\;\leq \;35$	5	0.00	5.00	0.00	5.00	0.00	5.00
SMGL	$D_{\text{mean}}\;\leq \;35$	5	69.03	0.00	68.98	0.00	68.77	0.00
SMGR	$D_{\text{mean}}\;\leq \;35$	5	35.51	4.80	34.32	5.00	35.11	4.96
ESO	$D_{\text{mean}}\;\leq \;40$	5	6.37	5.00	6.53	5.00	6.46	5.00
**Cumulative**	**Total**	**150**	**–**	139.03	**–**	141.53	**–**	**141.78**

**Table 18:** Summary of dosimetric performance across patients P6-P8 on 1^st^ rpCT: Manual (M), patient-specific DQN (Q), and patient-specific PPO (P). All dose metrics $\left(D_{\text{0\%/0.03cc/mean}}\right)$ in GyRBE. Bold values indicate superior dosimetry outcomes. Results for patients P1-P5 are summarized in Table 4.

Structure	Metric	P6			P7			P8		
		M	Q	P	M	Q	P	M	Q	P
CTV1	$V_{d_{Rx,{\text{CTV}}_{\text{1}}}}\;\geq \;98\%$	98.00	**98.01**	98.00	97.99	97.99	**98.00**	**98.01**	97.99	97.99
	$D_{0\%}\;\leq \;77$	**84.54**	84.74	85.85	**82.89**	84.47	83.44	**78.64**	82.44	84.30
CTV2	$V_{d_{Rx,{\text{CTV}}_{2}}}\;\geq \;98\%$	**98.69**	98.41	98.51	97.86	**98.06**	97.99	**97.63**	96.98	96.86
CTV3	$V_{d_{Rx,{\text{CTV}}_{3}}}\;\geq \;98\%$	**98.46**	98.15	98.39	**97.77**	97.51	97.51	**98.08**	97.16	97.30
BRS	$D_{0.03\text{cc}}\;\leq \;30$	1.11	**1.03**	1.23	**15.29**	15.94	15.74	23.07	**19.85**	20.23
SC	$D_{0.03\text{cc}}\;\leq \;30$	**45.13**	45.38	45.43	31.87	32.14	**31.85**	34.99	30.29	**30.27**
MAN	$V_{70\text{GyRBE}}\;\leq \;10\%$	**0.00**	**0.00**	**0.00**	**0.46**	0.52	**0.46**	**0.03**	0.07	0.21
LAR	$D_{\text{mean}}\;\leq \;40$	**0.00**	**0.00**	**0.00**	41.84	**38.99**	39.90	42.36	41.64	**40.61**
PHY	$D_{\text{mean}}\;\leq \;50$	**0.00**	**0.00**	**0.00**	**0.00**	**0.00**	**0.00**	50.47	**48.31**	48.50
PARL	$D_{\text{mean}}\;\leq \;26$	**13.20**	14.66	14.80	28.00	25.98	**25.86**	35.68	25.86	**25.61**
PARR	$D_{\text{mean}}\;\leq \;26$	**13.67**	14.13	14.31	17.32	**17.20**	17.31	23.26	**23.10**	25.32
COCHL	$D_{\text{mean}}\;\leq \;35$	**0.00**	**0.00**	**0.00**	**4.15**	4.40	4.37	20.38	**18.11**	18.43
COCHR	$D_{\text{mean}}\;\leq \;35$	**0.00**	**0.00**	**0.00**	**0.00**	**0.00**	**0.00**	7.54	**5.31**	5.60
SMGL	$D_{\text{mean}}\;\leq \;35$	36.19	**34.94**	34.97	69.03	68.98	**68.77**	67.71	**66.30**	66.60
SMGR	$D_{\text{mean}}\;\leq \;35$	39.59	35.01	**34.96**	35.51	**34.32**	35.11	35.91	35.35	**34.84**
ESO	$D_{\text{mean}}\;\leq \;40$	52.57	49.92	**49.69**	**6.37**	6.53	6.46	14.48	**12.24**	12.65
**Plan Score**	**(max=150):**	133.03	**135.24**	135.18	132.53	141.03	**141.35**	139.03	141.53	**141.78**

*Note*: OAR abbreviations - BRS: Brainstem, SC: Spinal Cord, MAN: Mandible, LAR: Larynx, PHY: Pharynx, PARL/PARR: Left/Right Parotid, COCHL/COCHR: Left/Right Cochlea, SMLG/SMGR: Left/Right Submandibular Gland, ESO: Esophagus.
